# Fortuitous discovery of a microfilaria of the genus Loa loa during a routine blood smear at the Hematology Laboratory of the Mohamed V Military Instruction Hospital in Rabat: a case report

**DOI:** 10.1099/acmi.0.000895.v3

**Published:** 2025-05-21

**Authors:** Mohammed Labrigui, Hamza Layat, Manal Bouikhif, Fatima Ziad, Zimi Khalil, Zineb Jerroundi, Hafid Zahid, Zohra Ouzzif

**Affiliations:** 1 Hematology and Immunohematology Laboratory, Mohamed V Military Instruction Hospital, Rabat, Morocco; 2 Faculty of Medicine and Pharmacy, Mohamed V University, Rabat, Morocco

**Keywords:** blood smear, filariasis, hyper eosinophilia, *Loa loa*

## Abstract

Loiasis is a parasitic infection transmitted by a vector, specifically through the bites of Chrysops genus tabanid flies. It is often associated with marked and persistent eosinophilia in affected individuals. We report the case of a 28-year-old Cameroonian male patient. His medical history includes an episode of malaria treated on an outpatient basis. As part of a diving internship in Morocco, the young serviceman underwent a medical fitness examination at the Medical Expertise Center for Aircrew Personnel of the Mohamed V Military Hospital, which included a biological assessment. This revealed a mildly elevated bilirubin level, lactate dehydrogenase activity at the upper limit of normal and eosinophilia at 1500 µl^–1^, without anaemia or thrombocytopaenia. A blood smear was prepared and stained with May-Grünwald Giemsa, revealing the presence of several small worms, with an appearance consistent with *Loa loa* microfilariae. This case of *L. loa*, identified in the haematology laboratory, is one of the rare diagnoses in Morocco. Therefore, biologists need to remain vigilant and carry out a thorough analysis of the blood count and blood smear.

## Data Summary

No data were generated during this research or are required for the work to be reproduced.

## Introduction

*Loa loa* filariasis is a cutaneous and blood-borne helminthiasis. It is strictly African, primarily found in equatorial and western regions. In medical literature, loiasis is also referred to as the ‘African eye worm’ due to one of its possible manifestations – the pathognomonic migration of the adult worm under the conjunctiva – although it often remains asymptomatic. Cases of loiasis have now frequently been reported in America, Europe, Australia and Asia due to importation [1].

This work reports a case of loiasis identified in a non-endemic area, Morocco. It also aims to highlight the importance of conducting and meticulously analysing a blood smear in the presence of eosinophilia, which can guide the diagnosis towards a parasitic aetiology.

## Case presentation

**Fig. 1.**
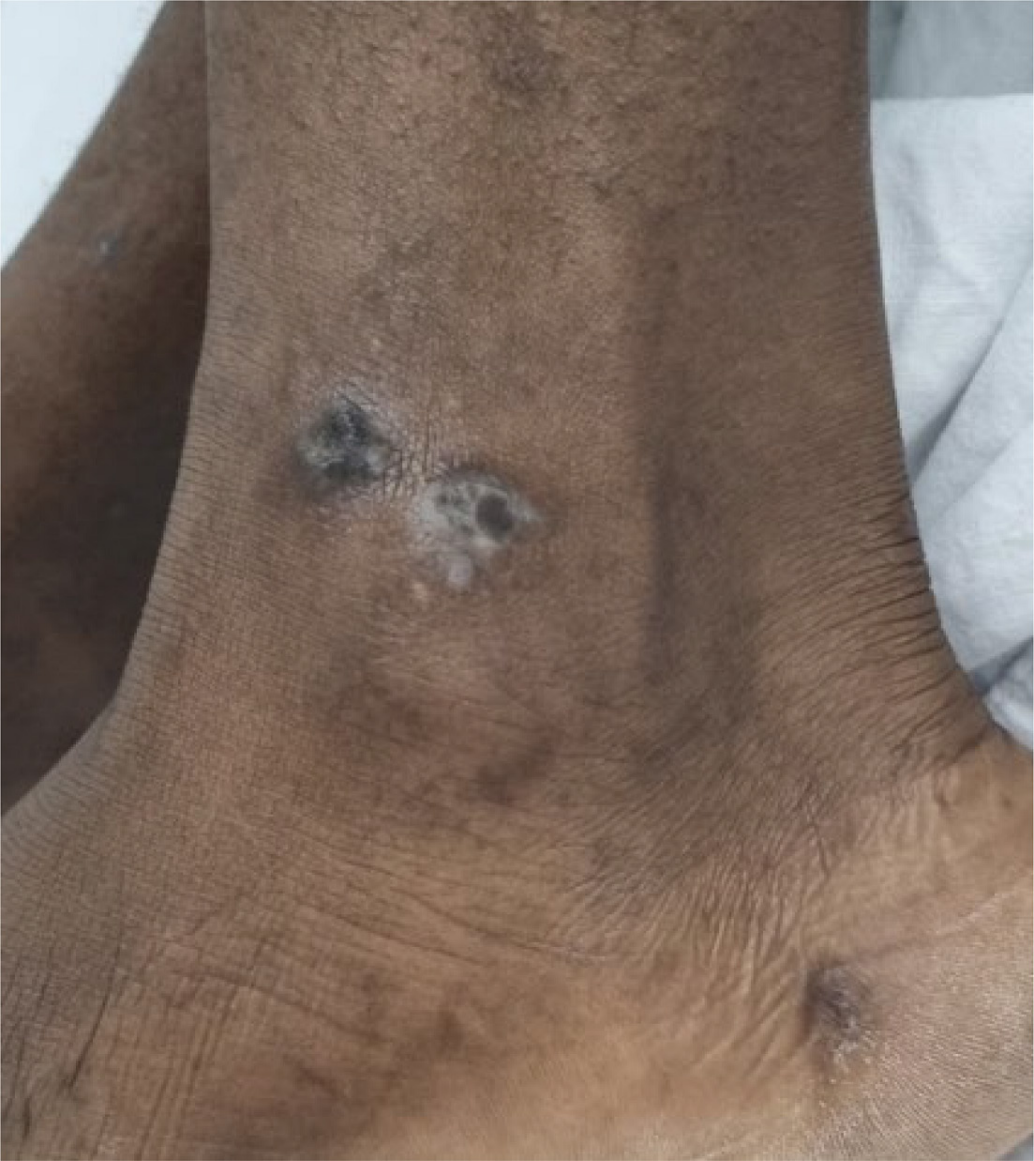
Skin nodules.

We report the case of a 28-year-old Cameroonian naval military personnel. His medical history includes an episode of malaria treated on an outpatient basis. The young serviceman underwent a fitness examination at the Centre for Medical Expertise of Aircrew at the Mohamed V Military Hospital (HMIMV). The clinical examination revealed no abnormalities except for two small, non-painful, not cellulitic skin nodules on both ankles (Fig. 1). A routine biological assessment revealed a slightly elevated bilirubin level at 14 mg l^−1^, a lactate dehydrogenase (LDH) activity at the upper limit (253 UI l^−1^), and eosinophilia at 1,500 µl^−1^, without anaemia or thrombocytopaenia. A blood smear was collected between 10:00 and 11:00 a.m., prepared for staining with May-Grünwald Giemsa (MGG) and examined at 100× magnification.

**Fig. 2.**
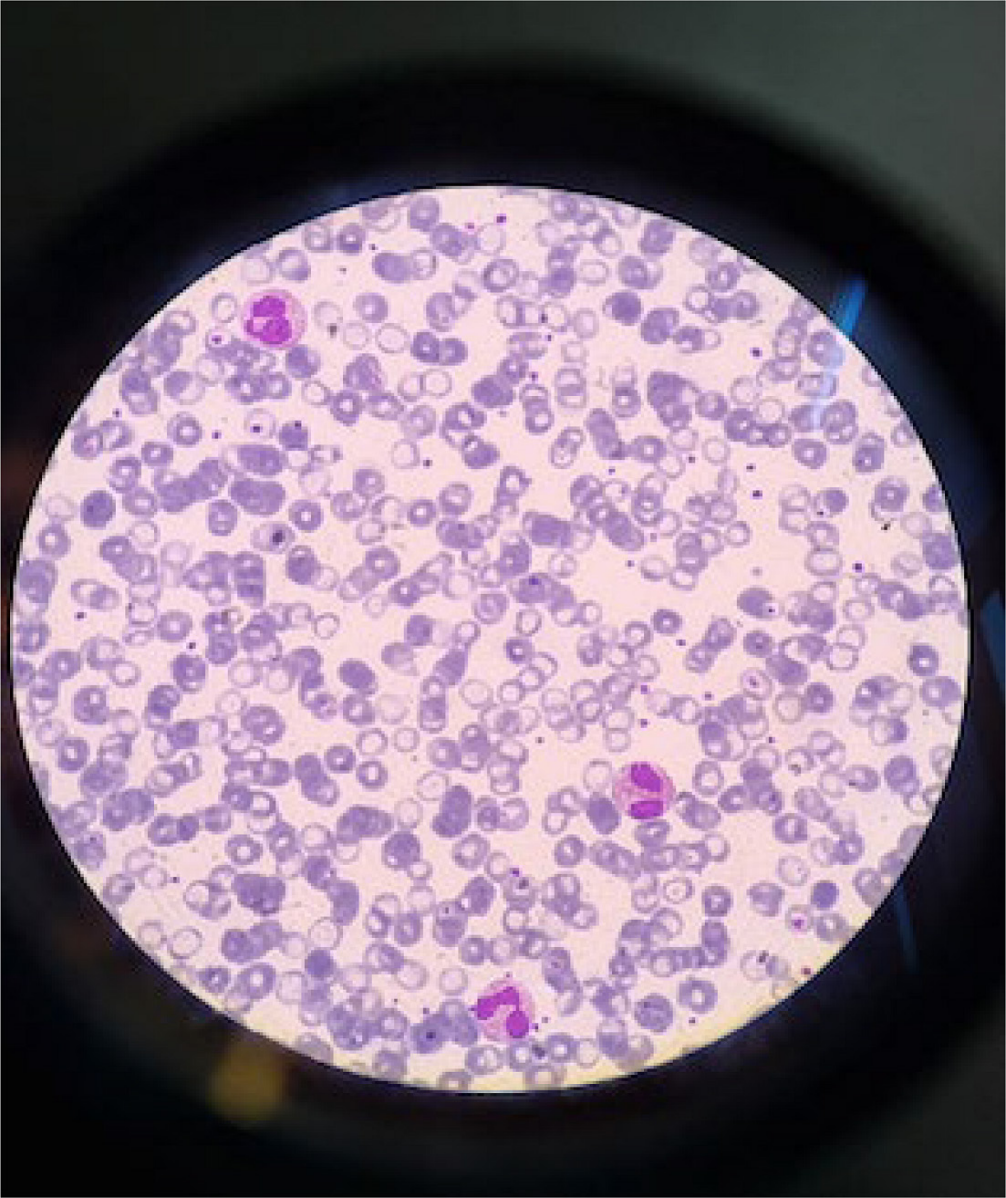
Eosinophils on a blood smear (× 100 objective, MGG staining).

The leucocyte formula showed evidence of 30% eosinophilic polymorphonuclear cells (Fig. 2), along with several small microfilariae approximately 100 µm in length.

**Fig. 3.**
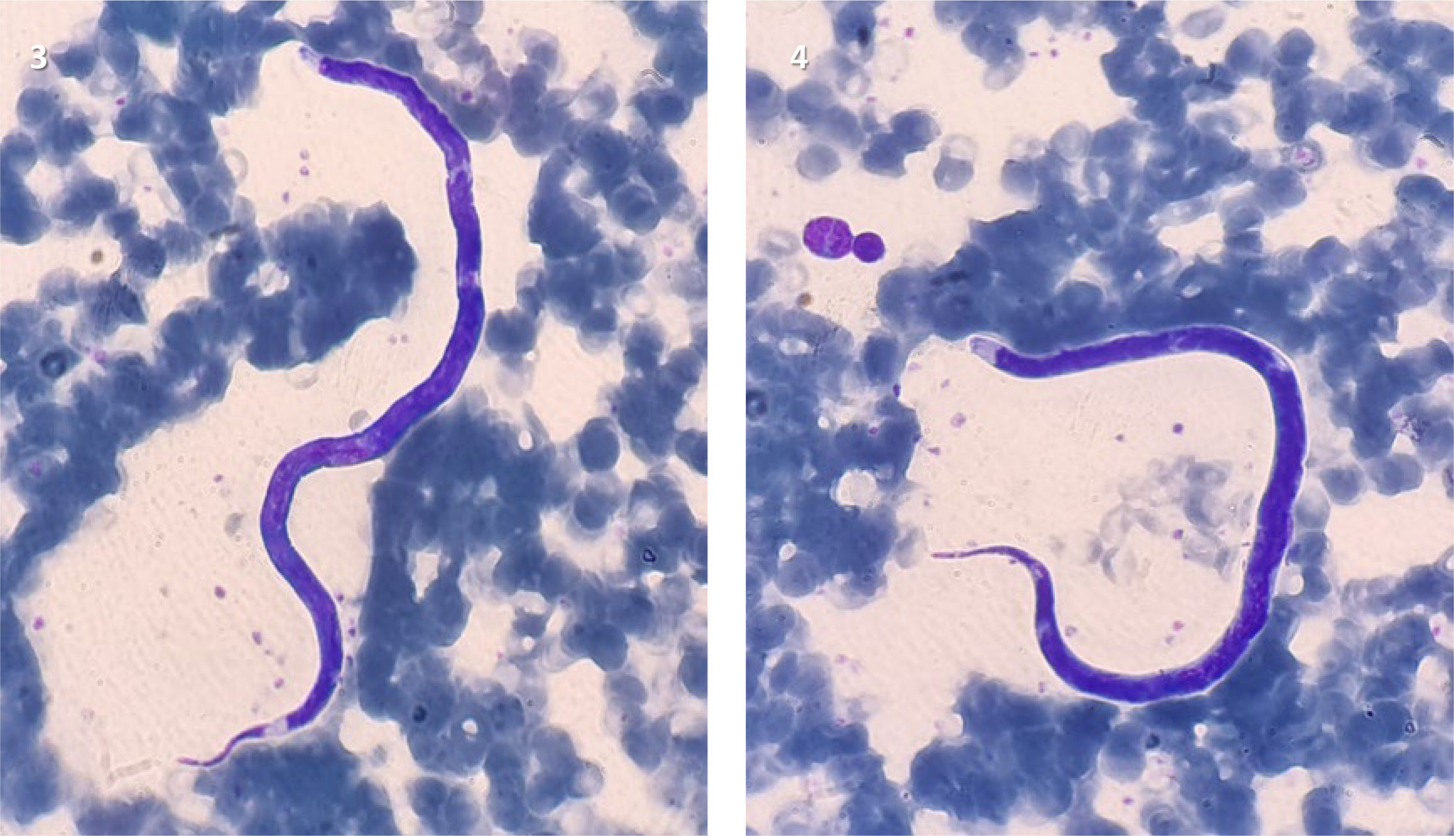
*L. loa* on a blood smear (× 100 objective, MGG staining).

These microfilariae have a short sheath not stained by MGG, a long cephalic space and a tapered caudal end (Fig. 3). Once the diagnosis of loiasis was confirmed, the patient was admitted to the infectious and tropical diseases department of the same institution for therapeutic management.

A complementary clinical examination did not reveal any additional signs beyond the two mentioned lesions, notably no limb oedema, signs of conjunctivitis or evidence of adult filarial worm under the conjunctivae. Ivermectin was prescribed for this parasitic infection; however, this medication is unfortunately not available in Morocco. The patient was transferred to a referral hospital in Cameroon for further management.

## Discussion

Loiasis is a vector-borne parasitic disease localized in Central Africa, caused by the nematode *L*. *loa*, transmitted to humans by the Chrysops fly. This parasitosis is frequently associated with significant and persistent eosinophilia [2]. It is endemic in ten African countries (Angola, Cameroon, Central African Republic, Chad, Republic of Congo, Democratic Republic of Congo, Equatorial Guinea, Gabon, Nigeria and Sudan), with an estimated prevalence of over 14 million individuals in hyperendemic areas [3].

The main clinical manifestations of loiasis include the subcutaneous Calabar swellings, the sub-conjunctival migration of the adult worm which can be fleeting and pose diagnostic challenges as reported by an American team [4] and pruritus. Sometimes, when the clinical picture is nonspecific, the diagnosis may take several years after the initial symptoms appear [5]. In this reported case, the patient, residing in Cameroon, an endemic country, presented no prior clinical manifestations. The *L. loa* parasite was discovered on his blood smear due to eosinophilia. This haematological anomaly is an important biological indicator guiding the diagnostic approach in the absence of an evocative clinical picture. The study by Spinello Antinori *et al*., conducted on 101 cases of loiasis imported into non-endemic countries in Europe and America, confirmed this and described eosinophilia in 82.1% of the patients [6].

These data should alert microbiologists, particularly in non-endemic countries like Morocco. Indeed, in the presence of eosinophilia in a patient from endemic countries, the microbiologist should consider preparing a blood smear to search for blood-borne parasites.

The treatment of filariasis is based on diethylcarbamazine, a synthetic derivative of piperazine. Gradually increasing doses of the medication are administered along with antihistamines or corticosteroids. However, post-treatment complications such as encephalitis may occur, particularly with ivermectin treatment. This molecule, used in an annual single-dose regimen, appears to be an excellent microfilaricide but is less effective against adult worms [7].

## Conclusion

This case of *L. loa* identified in the haematology laboratory of HMIMV is among the rarely diagnosed cases in Morocco. Given the strategic position of this institution at the national and international levels, and the participation of its personnel in overseas operations, biologists must be vigilant in meticulously analysing blood counts and smears. This ensures accurate diagnosis, appropriate patient management and the avoidance of complications from improperly conducted therapies.

## Abbreviations

HMIMV: Mohamed V Military Hospital
LDH: lactate dehydrogenase
MGG: May-Grünwald Giemsa
